# Policy on reintegration of women with histories of substance abuse: A mixed methods study of predictors of relapse and facilitators of recovery

**DOI:** 10.1186/1747-597X-2-28

**Published:** 2007-09-19

**Authors:** Nancy R VanDeMark

**Affiliations:** 1 Graduate School of Social Work, University of Denver, 2148 South High Street, Denver, CO 80208 USA

## Abstract

**Background:**

The predominant U.S. policy approach toward individuals with substance abuse problems has relied on stigma and punishment by withholding access to education, cash assistance, housing, social support, and normal social roles. In contrast to this approach, the theory of reintegrative shaming asserts that providing individuals with the opportunity to reconnect with society is more effective in reducing potential to relapse to crime and drug abuse. Strategies that promote such reconnection include expanding access to basic needs and supportive relationships along with increasing opportunities to fully participate in mainstream social roles.

**Methods:**

The present cross-sectional study examined the predictors of relapse and the facilitators of recovery in a sample of 325 women with histories of substance abuse. Analysis of secondary data, collected as part of a national cross-site study, employed a mixed methods approach conducting (1) logistic regression to examine the predictors of relapse and (2) an inductive qualitative analysis of responses from open-ended items to explore the women's perceptions of barriers to and facilitators of recovery.

**Results:**

Results suggest that lower levels of instrumental support, affective support, and participation in normal roles (such as parent, employee, student, and citizen) are significant predictors of relapse to drug use and criminal behaviors. Qualitative findings support the quantitative results, revealing that participating women perceived the variables of support and role participation as critical in facilitating their recovery. They also noted the importance of individual characteristics such as optimism and strength and emphasized the significance of their relationship with their children in motivating them to avoid relapse. Findings suggest that punitive policies toward women with substance abuse histories may be ineffective.

**Conclusion:**

The author concludes that current policies designed to withhold access to basic needs such as housing, education, cash assistance, and positive relationships may deprive women with histories of substance abuse of the means to reconnect with society. Policies that promote access to basic needs and offer avenues for women to participate in normal societal roles should be more fully explored.

## Background

Substance abuse is a social problem of great importance to contemporary American society. According to the National Survey on Drug Use and Health, it is estimated that 22.5 million people, or 9.4% of the U.S. population, experience problems with substance abuse [1]. Substance abuse places a considerable burden on society: Hundreds of billions of dollars are spent each year to reduce the influx of drugs, to provide treatment to substance abusers, and to deal with consequences of substance abuse [2].

### Substance abuse policy

U.S. policy tends to view substance abuse as a moral weakness and to stress punishment. This view of substance abuse is reflected in the current 2:1 ratio of spending on interdiction and criminal sanctions as compared to treatment, prevention, and research [2]; the widespread use of mandatory sentencing for drug offenses; and the abundance of public policies that punish individuals with histories of substance abuse [3-8].

Punitive policies toward substance abuse have been particularly evident in the past three decades. Although the concept of a "War on Drugs" first emerged during the Nixon administrations (1969–1974), the 1980s and 1990s were characterized by dramatic policy change and spending increases related to drug use reduction. These policies included mandatory sentencing for drug offenders; "one strike, you're out" or "zero tolerance" policies in public housing; restrictions on education loans, cash assistance, and food stamps for drug offenders; and elimination of Social Security benefits for individuals with drug-related disabilities [3,4,7,8].

Between 1980 and 1997, drug arrests (largely of individuals charged with drug possession) tripled, with drug offenders making up more than half of the U.S. prison population [9]. Incarceration of women also skyrocketed; drug offenses accounted for nearly half (49%) of this increase [10]. These increased incarceration rates have been attributed in part to policy shifts related to the War on Drugs – in particular, the emphasis on mandatory drug sentencing [7].

In addition to the War on Drugs, during the past twenty years a series of policies barring substance abusers from public housing have been implemented. One provision of the Anti-Drug Abuse Act (1988) required public housing authorities to evict tenants when they or their families were found to have engaged in criminal activity [5,11]. In 1990 these laws were strengthened, resulting in what has been called the Federal government's "one-strike" policy. The one-strike policy required housing authority officials to place individuals who had engaged in criminal activity on or near public housing sites, or had guests who had engaged in these behaviors, at the end of waiting lists for housing. Thus, the policy often prevented individuals with a history of drug possession, and those with family members who had engaged in drug-related activities, from obtaining housing. In 1998, Federal policy recommending that local authorities deny housing if a household member had a drug-related criminal history was passed [5,12]. In effect, all drug abusers were categorized with sex offenders and methamphetamine manufacturers as exceedingly dangerous to the health and well-being of other public housing residents.

Policies restricting educational benefits to people with histories of substance abuse have also been widespread in recent years. In 1998, Congress amended the Higher Education Act to include provisions denying Federal education assistance to individuals convicted of drug charges [12,13]. Although in recent years steps have been taken to repeal these provisions, as recently as the summer of 2005, proposed amendments upheld the denial of financial aid to students convicted of drug possession while enrolled in school [12,13].

The Adoption and Safe Families Act (ASFA) of 1997, created to address the problem of growing foster care rolls, produced additional hurdles for parents with substance abuse problems who were involved with the child welfare system [14,15]. ASFA established time limits for family reunification and created an expedited process of permanency planning [14,15]. The provision having the most profound impact on substance-abusing families was the timetable for termination of parental rights [14,15]. Under this provision caseworkers were required to begin permanency planning immediately following removal of the child from the home [14]. Yet a study conducted by the Child Welfare League of America [16] concluded that child welfare agencies were unable to provide substance abuse treatment to two-thirds of the families that needed it. Without the increased availability of treatment, along with close coordination between substance abuse and child welfare systems, substance-abusing families were faced with unnecessary separations, permanent loss of custody, and needless suffering [12,15,16].

Clearly, substance abuse is a costly and important social policy issue that has captured a great deal of attention and resources during the past quarter century. Few social problems have such a widespread effect on both individuals and society. Despite the emphasis of U.S. policies on punitive approaches, responses such as mandatory minimum sentencing and bans on public benefits have been widely criticized by policy researchers as ineffective in addressing the problem of drug abuse [8,17].

### Reintegrative shaming theory

In contrast to the predominant U.S. policy approach to substance abuse, criminal justice theorist John Braithwaite proposes the theory of "reintegrative shaming," [18-21] which asserts that stigmatizing and punitive policy approaches are not only ineffective in addressing socially unacceptable or illegal behaviors, they actually reinforce and perpetuate these problems. Braithwaite contends that the manner in which individuals who have violated social norms are treated dictates their propensity to repeat unacceptable behaviors. He argues that communicating social disapproval (shaming) is necessary to discourage crime, but that when social disapproval is accompanied by opportunities to rejoin society, people are less likely to perpetuate unacceptable behaviors. He describes the two contrasting approaches as "reintegrative" versus "stigmatizing."

Stigmatizing approaches communicate disapproval in ways that are disrespectful and promote humiliation. Further, they provide no avenues for individuals to rejoin society [18]. Policies that harshly label people and block access to acceptable social roles are examples of stigmatizing approaches. In contrast, reintegrative approaches acknowledge unacceptable behaviors but follow with forgiveness and provide avenues to reconnect with society. The central feature of reintegration is reconstruction of attachment between the offender and the community through relationships of mutual dependence [19,20,22].

Interventions based on the theory of reintegrative shaming have been explored empirically with adult and adolescent offenders, showing mixed but promising results. These findings suggest that the combination of clear messages that a behavior is wrong, coupled with opportunities to be forgiven and to recreate social relationships, reduces subsequent recidivism [23-25]. Further, findings from these studies suggest that policy approaches promoting reconnection to relationships of mutual dependence may be more effective in reducing relapse to crime and drug abuse than punishment or benefit bans.

### Social support and participation

Allied with Braithwaite's claims, research in substance abuse treatment demonstrates the potential power of supportive relationships in sustaining recovery from substance abuse. For example, studies suggest that social support, including participation in peer support groups, predicts decreased alcohol consumption and increased abstinence in substance-abusing populations [26-32]. However, these studies have largely investigated the influence of receiving support from others, rather than both giving and receiving support or engaging in relationships of mutual dependence as advocated by Braithwaite. One exception is the research conducted on 12-step programs such as Alcoholics Anonymous that promote the concept of helping others as a path to recovery. This research has demonstrated relationships between participation in 12-step groups and subsequent lower levels of subsequent substance abuse [33,34]. Further, one study found that substance-dependent people who were engaged specifically in helping others were significantly less likely to relapse during the year following treatment [35], suggesting that participation in meaningful and responsible social roles may play a role in recovery from substance abuse. Additional investigation is needed to clarify the relationship between the mutual dependence associated with participation in normal societal roles and substance abuse recovery.

### Instrumental support

In addition to social support and participation, evidence points to the importance of instrumental support (i.e., access to necessities such as an adequate and stable source of income, safe and stable housing, and employment) as a critical element in recovery from substance abuse. Research has established an association between financial stress (including lower income) and poorer substance abuse and criminal justice outcomes [31,36,37]. Furthermore, substance abuse treatment programs that address basic needs such as medical care and housing have been found to be more effective than programs that do not address these needs [38]. This finding suggests that instrumental support, in addition to social support, may be critical to ongoing recovery from substance abuse.

The present study uses quantitative methods to explore Braithwaite's contention that providing avenues to reconnect with society – including means to support oneself and one's family, support from others, and access to meaningful social roles – decreases the propensity to relapse. Further, it employs qualitative methods to provide insight into how these predictors are perceived by a sample of women with histories of substance abuse.

## Methods

### Design

This cross-sectional study relied on secondary analysis of data from the Women with Co-occurring Disorders and Violence (WCDV) study, a national multi-site study funded by the U.S. Department of Health and Human Services' Substance Abuse and Mental Health Administration from 2000 through 2003 [39].

The WCDV study evaluated the effectiveness of integrated services for women affected by substance abuse and mental illness who were also victims of violence. This longitudinal, quasi-experimental study included 2729 women who entered mental health or substance abuse treatment over a two-year period at nine sites across the U.S. Follow-up interviews assessing outcomes were conducted with enrolled women six and twelve months following the initial interview. To be eligible for the study, women were required to (1) have a current diagnosis of either substance abuse/dependence or an Axis I mental health diagnosis, (2) to have experienced both problems in the past five years, and (3) to have experienced violence in their lifetimes. The primary findings from the WCDV study can be found in other publications [40-42].

Because responses to open-ended questions were available for the entire dataset, and these responses were anticipated to provide information on key variables of interest (as well as to offer context for the quantitative analysis), the present study employed a mixed methods design. Since a de-identified dataset was used, an Institutional Review Board exemption was requested and obtained.

### Sampling

Of the 2729 women in the original study, 813 were determined to have a significant drug problem at the time of entry into the WCDV study, as assessed by the Addiction Severity Index Fifth Edition [43], and to have completed a follow-up interview 12 months later. Results of an a-priori power analysis suggested that 325 cases would likely yield adequate power (.80) to detect small effects of the independent variables on the dependent variable with alpha set at .05. Because the mixed methods study design required analysis of two open-ended items and resources were limited, a sample of 325 cases was randomly selected from the pool of eligible cases using the random case selection feature in the Statistical Package for the Social Sciences.

### Variables

In order to reduce the number of independent variables and thus increase the power of the statistical tests performed, composite variables were constructed to represent the three major factors hypothesized to predict successful reintegration into society.

### Instrumental support

Instrumental support included items related to a woman's ability to provide basic support for her family. Items included in the variable "instrumental support" are listed in Table 1. The categorical item assessing housing status was recoded into a dichotomous variable indicating whether the individual was residing in stable housing versus living on the street or in a shelter. When necessary, items were reverse coded so that a more negative status was assigned a higher numerical score. Each item was assumed to be independent, was given equal weight in the construction of the composite variable, and was divided by the total number of response categories and then summed to create a composite score.

**Table 1:** Items Included in Composite Independent variables

Composite Variable	Question
Instrumental Support	In the past 30 days, where have you been living most of the time? (dichotomous variable with housed = 0, homeless = 1)
Instrumental Support	In general, how safe do you feel where you are living now? (scale of 1–5 with extremely safe = 1, not at all safe = 5)
Instrumental Support	In the past six months, have you had serious money problems, for example, not enough money for food, clothing or rent? (dichotomous variable with no problems = 0, problems = 1)
Affective support	Have you been physically abused – for example, hit, choked, burned or beaten or severely punished – for example, locked up, shut in a closet, tied up or chained by someone you knew well such as a parent, sibling, boyfriend or girlfriend within the past six months? (dichotomous)
Affective support	In the past six months, have you been discriminated against in a way that was highly distressing because of your race, ethnic group, gender, sexual orientation, or religion? (dichotomous)
Affective support	In the past six months, how often has there been someone known well by you, who has made you feel unsafe? (scale of 1–4)
Affective support	In the last six months, have you participated in any type of peer support or self help services? (dichotomous)
Affective support	During the past week to what extent have you been experiencing never feeling close to another person? (scale of 1–5)
Affective support	Responses to the open-ended question – What has been helpful in your healing and recovery? – identifying support of family members and friends (other than recovering peers). (coded open-ended responses as dichotomous with no support mentioned coded as 1 and any support mentioned coded as 0)
Participation – Normal Roles	Are you currently in school? (dichotomous)
Participation – Normal Roles	Are you currently employed? (dichotomous)
Participation – Normal Roles	Of those children under 18, how many live with you? (dichotomous)
Participation – Normal Roles	Responses to the open-ended question – What has been helpful in your healing and recovery? – identifying the importance of participation in family, community or helping others. (dichotomous with no mention of the importance of these items coded as 1 and mention coded as 0)
Participation – Autonomy	In the last six months, have your parental rights been terminated? (dichotomous)
Participation – Autonomy	At this time, are you required or court ordered to participate in substance abuse or mental health treatment? (dichotomous)
Participation – Autonomy	In the past six months, have you been separated from your child against your will? (dichotomous)

### Affective support

The affective support variable comprised items that reflected the quality of support from friends or family. The variable "affective support," which included items listed in Table 1, reflected the quality of participants' personal relationships. As necessary, items were reverse coded so that a higher score depicted a less positive status; then, a new variable was created for each item by dividing the score by the number of possible response categories. This enabled each item included in the composite score to be equally weighted. To create a composite variable depicting affective support, the weighted variables were summed.

### Participation

Finally participation included items that suggested a woman's responsibility to fulfil meaningful social roles and having control and autonomy over her own life. Items included in the normal social role variable are listed in Table 1. The composite variable "normal roles" was derived by dividing each response by the number of response categories for each included variable, and then summing all of the variables together. This created a composite variable that weighted all items equally.

The variable "autonomy" reflects the freedom to make choices and decisions about one's own life; included items are listed in Table 1. All items were coded with a 1 indicating the presence of the event and a 0, the absence of the event. Thus, a higher score indicated a less desirable status. Each item was divided by the number of response categories and then summed to create a composite variable. This strategy gave equal weight to each variable in the calculation of the composite score.

### Relapse

A composite variable representing constructs commonly associated with relapse – illegal drug use and other criminal behaviors – was developed and included the following items:

• In the past 30 days, how many times have you been arrested? (number of arrests)

• In the past 30 days, did you receive income from illegal sources? (dichotomous)

• In the past six months, have you ever had sex when you did not want to in exchange for money, drugs, or other material goods such as shelter or clothing? (dichotomous)

• How many days in the last 30 have you used illegal drugs? (number of days)

• In the past thirty days, have you injected drugs? (dichotomous)

Relapse was defined as any affirmative report of an illegal behavior listed above; therefore, any response indicating that an individual had engaged in any of the activities was coded with a 1 and any negative response was coded with a 0. A Cronbach's alpha that included the five items in the dependent variable was conducted and demonstrated moderately high internal consistency (.62). The five dichotomous variables were later summed to create the composite variable "relapse."

### Control variables

Age was introduced as a covariate to control for the possibility that the effect of the predictor variables on the dependent variables was a function of people "maturing out" of illegal behaviors. Other covariates included ethnic/racial minority (White versus non-White) status and criminal history (determined by the respondent's answer to "Have you ever been in jail or prison?"). Finally, substance abuse treatment history in the 30 days prior to the interview was included as a covariate, to ensure that the protected environment of treatment was not exerting influence over the relapse to drug use or other illegal behaviors.

### Characteristics of the sample

The average age of the women in the sample was 36.2 years (*SD *= 8.9) with ages ranging from 19 to 66 years. Nearly half of the women (48.8%) reported not having completed high school. The women reported receiving an average of $948 (*SD *= 852.3) per month in income; about one-third (31.4%) were employed either full or part time. Of the 323 women in the sample who provided information about ethnicity, approximately one-fifth (20.1%) reported being of Hispanic or Latina decent. Of those reporting race, 57% were Caucasian, 22% were African American, 9% were American Indian, 1% were Asian, and 17% were other or mixed race. The majority of the women in the sample (87.7%) reported having children, with 83.7% having children under the age of 18 years.

Women reported a variety of physical, emotional and behavioral histories and current problems. A large majority of the sample (83.4%) had been incarcerated, with 9% reporting incarceration in the six months preceding the interview. The women in the sample had experienced many different types of abuse. Eighty-four percent reported having been emotionally abused, 30.5% reported a history of physical neglect, 82.7% reported physical abuse, and 62.7% reported a history of sexual abuse.

### Qualitative data analyses

An inductive approach to qualitative data analysis was used to examine the responses to the two open-ended items (What helped in your healing and recovery? and What hurt your healing and recovery?). The data were assigned open codes that emerged from the data (as contrasted to codes generated from theory). Later, axial codes were assigned representing themes found in the data. Of the 325 cases in the sample, 103 were randomly selected for check-coding by a second researcher. Check-coding was completed initially as a tool to develop the coding structure and again to validate the application of the coding structure. Throughout the qualitative data analysis process, the two researchers discussed themes emerging from the data as well as interesting and perplexing questions arising from the data analysis.

To quantify responses to the open-ended items used in the quantitative analysis, themes that corresponded to social support and participation were identified. A dichotomous variable for each relevant theme was created and cases where the participant mentioned the presence of a particular variable were coded with a 0. If the participant failed to mention a particular variable as important to recovery, a 1 was entered for that variable.

### Quantitative data analyses

Following development of the composite variables, descriptive statistics including frequencies and measures of central tendency and dispersion were examined on all dependent, independent, and control variables. Table 2 displays the percentages, means, ranges, and standard deviations for the independent, dependent, and control variables.

**Table 2:** Means, Percentages, Standard Deviations and Range for Independent, Dependent, and Control Variables (N = 325)

Variable	χ¯ MathType@MTEF@5@5@+=feaafiart1ev1aaatCvAUfKttLearuWrP9MDH5MBPbIqV92AaeXatLxBI9gBaebbnrfifHhDYfgasaacH8akY=wiFfYdH8Gipec8Eeeu0xXdbba9frFj0=OqFfea0dXdd9vqai=hGuQ8kuc9pgc9s8qqaq=dirpe0xb9q8qiLsFr0=vr0=vr0dc8meaabaqaciaacaGaaeqabaqabeGadaaakeaacuaHhpWygaqeaaaa@2E7B@ or %	SD	Minimum Score	Maximum Score
Instrumental Support	.70	.37	.20	2.00
Affective Support	1.47	.63	.45	3.80
Participation – Autonomy	.44	.67	.00	2.00
Participation – Normal Roles	1.55	.43	.50	2.00
Relapse	25.39	-	.00	1.00
Age	36.19	8.88	19.00	66.00
Criminal history	83.64	-	.00	1.00
Ethnicity	47.83	-	.00	1.00
Treatment history	41.35	-	.00	1.00

Correlations were performed on the independent and control variables to examine univariate multicollinearity and assist in interpretation of multivariate findings. Correlations ranged from .00 to.36, indicating small to moderate correlations. Table 3 lists correlations among the independent and control variables.

**Table 3:** Intercorrelations among Independent and Control Variables (N = 325)

Variable	1	2	3	4	5	6	7	8
1. Instrumental Support	1.00	.36	.12	.30	.11	.00	-.03	.02
2. Affective Support	-	1.00	.00	.28	.12	-. 03	-.04	-.14
3. Participation – Autonomy	-	-	1.00	.16	-.14	-.08	.07	.21
4. Participation – Normal Roles	-	-	-	1.00	.28	-.01	-.02	.09
5. Age	-	-	-	-	1.00	-.08	.00	.02
6. Criminal History	-	-	-	-	-	1.00	.02	-.03
7. Minority Ethnicity	-	-	-	-	-	-	1.00	.06
8. Treatment Status								1.00

To test the hypothesis that lower levels of the combined independent variables of instrumental support, affective support, and participation in normal roles and autonomy would predict relapse, sequential logistic regression was used. A linear regression to check for multivariate multicollinearity was performed and demonstrated acceptably low levels of multicollinearity among independent and control variables.

A sequential logistic regression was performed to examine the relationship between the independent variables and the dichotomous dependent variable (relapse). This sequential approach was used to examine the contribution of the independent variables (instrumental support, affective support, and two forms of participation: normal roles and autonomy) after controlling for age, minority status, criminal history, and current treatment status. The logistic regression introduced the four control variables in the first block and then each independent variable (instrumental support, affective support, and the two types of participation) in separate blocks.

## Results

### Qualitative findings

The 325 women in the sample were asked to respond to two questions: What helped in your healing and recovery? and What hurt your healing and recovery? Analysis of their responses yielded a number of themes related to the women's perceptions of the factors shaping their ability to recover from their substance abuse problems. Table 4 specifies the number of times each theme was mentioned in response to either of the open-ended questions.

**Table 4:** Number of Mentions of Themes Emerging in Response to Open-ended Questions (N = 325)

Theme	Number of mentions
Treatment providers and treatment activities	175
Psychological characteristics	127
Relationship with children	111
12-step program participation	99
Instrumental support	95
Support from family and friends	95
Acknowledging and accepting past	86
Coercion into services	72
Reliving painful experiences	37
Participation in family and community	23

### Treatment providers and treatment activities

Women described their treatment experiences and the qualities of treatment providers as playing an essential role in recovery. They noted that helpful providers had characteristics such as patience, a non-judgmental attitude, and a sense of humor.

[My counselor is ] very understanding – very funny. She just has a way of looking at things that makes everything positive.

Further, women stated that providers who unnecessarily flaunted their power and who were judgemental were destructive to recovery efforts.

### Psychological characteristics

Many of the women also described individual psychological characteristics, such as a positive attitude, that were critical to their recovery. They also discussed the importance of standing up for themselves, having faith in their own ability, and keeping a positive vision for the future.

[What is helpful to my recovery is] that I care for me today – I am somebody. I'm not a people pleaser, I am a go-getter. I do have dreams – bottom line I have goals to accomplish.

### Relationships with children

Many participants discussed how their relationships with their children helped motivate them to recover, yet the separation from their children both hurt them and was harmful to their recovery. They reported that knowing their children were safe while they were in treatment helped them concentrate on recovery. More importantly, they noted that their desire to provide a better life for their children served as a primary motivator for recovery.

Keeping focused on getting my children back [helps my recovery]. Being able to teach them how to love and how to survive in life.

### Twelve-step program participation

Participants mentioned the importance of 12-step meetings, and the principles of helping others and giving back the help they had received, as important contributors to their recovery. Some participants also mentioned that their failure to participate in 12-step meetings interfered with their ability to sustain recovery.

The support of my NA program and my sponsor [has been helpful]. It gives me somewhere to go to share and learn. It gives me the opportunity to meet people like myself. Doing volunteer work with the NA program is very rewarding for me.

### Instrumental support

Women identified having financial assistance, an opportunity to save money, and help in finding a job as critical factors in their recovery. Conversely, they discussed how losing a job and not having access to adequate financial resources hindered their ability to be independent and to care adequately for their children. A few women also discussed the double bind they experienced with the child welfare system when appropriate housing was not available or a criminal record prevented access to housing.

Not having the resources I need hurts my recovery. I can't get housing because of my [criminal] record. Can't get my kids unless I have housing. Can't get transitional assistance unless I have my kids. Even though I have [a diagnosis of] bi-polar [disorder], I can't get disability because of my substance abuse. I can't get to meetings because I have no transportation. I exhausted my program options.

### Support from family and friends

Receiving support from someone who believed in their ability to recover and who provided unconditional love despite relapses was identified by the participants as essential for recovery. In particular, some women described the importance of having people believe in them even when they may not have full confidence in themselves.

My children have been helpful to me. I've explained to them what I've done – what my problems are, and they've been very understanding about that. And they just try to keep encouraging me to do better.

### Acknowledging and accepting the past

Participants found value in acknowledging past experiences of abuse and trauma and their relationship to current life challenges. Further, they described the importance of being truthful with themselves about their mistakes and accepting and forgiving themselves. They noted that the guilt and shame about the way they had treated others – in particular, about not properly caring for their children – presented the most difficult area for self-forgiveness.

The most hurtful thing is having to live with the guilt of what I did. The choices I made and having to live with that. Forgiving myself, that's been really hard...Knowing it took time to get my kids and I back together, knowing the pain I caused them. I had to be really strong because I couldn't see my children knowing the pain I caused them.

### Coercion into services

Some participants who were required by the courts or social services to seek treatment found the requirement helpful. In addition, some believed that serving time in jail helped them acknowledge their problems. Other women reported that the coercion of child welfare hurt their recovery, and stated that the loss or potential loss of their children actually promoted relapse and interfered with recovery.

I don't have my son; it's hard to stay clean not knowing if he's coming back. It's hard to deal with the anxiety.

### Reliving painful experiences

Participants described the value of understanding the relationships between their past experiences of abuse and trauma and their current problems. They acknowledged that reliving these experiences was exceptionally painful and often these experiences hurt their recovery but many of them also noted that reliving the memories was important to understand their current life challenges.

Realizing the depth of the destruction of my life [was hurtful]. Dealing with the grief. Coming to terms with the trauma I've lived through. I know I had to go through all that to be where I am today.

### Participation in family and community

Women described the importance of roles and relationships that required them to act responsibly and provide mutual support (e.g., holding a job, supporting other family members, and engaging in volunteer work). Some of the participants in particular described the importance of having children who look to them to be role models and discussed how this motivated them to be better people.

[It's] helpful to look at the relationship with my young daughter cause she needs me. It gives me the power to want to change.

### Quantitative findings

The sequential logistic regression model including only the four covariates was not significant, χ^2 ^= 6.14, *df *= 4, *N *= 313, *p *= .189, indicating that the covariates did not significantly predict relapse. A test of the full model including the four covariates and four independent variables did yield a significant model, χ^2 ^= 54.65, *df *= 8, *N *= 313, *p *< .001; therefore, the test of the four independent variables, not including the four covariates, yielded a significant model, χ^2 ^= 48.51 *df *= 4, *N *= 313, *p *< .001. No significant relationships between interactions of the independent variables and the covariates and relapse were found.

Examination of the univariate contribution of each variable revealed that instrumental and affective support and participation in normal roles significantly contributed to the prediction of relapse. The odds ratios indicated that with all the variables in the model, as levels of instrumental and affective support decreased, women were twice as likely to report relapse; as normal roles decreased, they were nearly four times as likely to report relapse. The participation variable of autonomy did not emerge as a significant univariate predictor when all the other independent variables were in the model. The odds ratios are found in Table 5.

**Table 5:** Logistic Regression Predicting Relapse from the Combination of Independent Variables (N = 325)

Variable	*B*	*SE*	*Odds ratio*	*p*	*95% CI for Odds Ratio*	
					*Lower*	*Upper*
Age	-.01	.02	.99	.40	.95	1.02
Criminal history	-.59	.37	.56	.11	.27	1.14
Ethnicity	-.06	.29	.95	.84	.54	1.66
Treatment history	-.49	.31	.61	.11	.34	1.12
Instrumental support	.98	.40	2.65	.02	1.21	5.81
Affective support	.70	.23	2.01	.01	1.27	3.18
Participation – Autonomy	.14	.22	1.15	.52	.75	1.75
Participation – Normal Roles	1.35	.42	3.85	.01	1.68	8.80

*Note*. The odds ratios were tested using large sample z-approximations.

## Discussion

The results of both the quantitative and qualitative analyses lend support for Braithwaite's [18-20,22] assertion of the effectiveness of reintegrative approaches that allow women with histories of substance abuse to reconnect with society. The quantitative findings suggest that women who lack access to housing and financial stability, support from others, and mainstream social roles are more likely to relapse than women who have access to these elements. Although the quantitative findings do not suggest that punitive policies cause relapse, the associations offer that at a minimum, punitive policies are not effective in decreasing the propensity to relapse. Further, the qualitative findings provide insight into participants' views about the relationship between withholding opportunities and relapse. Women cited many of the current punitive policies, such as withholding housing and cash assistance and removing women with histories of substance abuse from the parenting role, as significant barriers to recovery. They further suggested that the opportunity to assume normal roles such as student, employee, and parent contributed positively to their potential for recovery.

Interestingly, no relationship between the participation variable of autonomy and relapse was found. The present study relied on the hypothesis that having control over one's life (the absence of coercion) was an important characteristic of full participation in society; however, the women's qualitative responses indicated that coercion had mixed benefits in recovery. While some women reported that being coerced by courts helped them accept treatment and become focused on priorities, others reported that being threatened with the loss of their children distracted them from recovery. It may be that coercion associated with loss of child custody has a different effect on relapse than potential loss of personal freedom.

One rationale for employing mixed methods in this study was to identify intervening variables that were not accounted for in the quantitative analysis, but would point to the next steps in investigation. The qualitative analysis suggests that in addition to the independent variables explored in the quantitative analysis, individual psychological qualities or characteristics may factor into the propensity to sustain recovery. For example, women in the sample described the importance of a positive vision for the future, assertiveness and self-confidence, readiness to make needed changes, willingness to trust and accept help from others, and faith or spiritual connections. Additional study of the psychological characteristics of women who are able to transition to recovery could help in crafting policies that recognize and bolster positive qualities such as self-confidence and willingness to change and possibly match policy interventions with individual psychological characteristics.

### Limitations

This study has a number of strengths and weaknesses in its contribution to the scientific literature on substance abuse policy. Its major weakness is its inability to speak to the causal relationship between instrumental and social support, participation in normal roles, and propensity to relapse. Nevertheless, the negative association between these variables and relapse counters assumptions underlying much of existing substance abuse policy, which presumes that withholding support and access to normal roles prevents drug abuse and other illegal behaviors. In this regard, the findings presented here move the literature one step further in questioning these presumptions, and also suggest that future research examine the possibility of a causal relationship between these variables and relapse.

Additionally, the data used in these analyses were self-reported; as a result, it is possible that women over- or underreported their criminal behaviors or drug use out of a desire to please the interviewer or fear that the information would be shared with criminal justice or child welfare agencies. To minimize this concern, the original study data were collected by researchers, not clinicians, and participants were assured that no information would be shared with outside sources. Further, a Federal certificate of confidentiality was secured to ensure that the data would not have to be released by court order. Nonetheless, the impact of social desirability on the validity of these self-reported data is unknown.

Another limitation of this study is its focus on a somewhat specialized sample of substance-abusing women with histories of both mental illness and violence, who chose to enter substance abuse or mental health treatment. Although their histories of mental health problems and trauma limit the generalizability of the findings, the problems of mental illness and trauma are very common among women seeking substance abuse treatment. Studies of women entering treatment for substance abuse suggest that over one-fourth have experienced physical abuse [44-47], and over half have a coexisting mental health disorder [48,49]. Hence, the experiences of the women in this sample are likely shared by a large proportion of women entering treatment for substance abuse. Nevertheless, generalization to groups of non treatment-seeking people and treatment-seeking men should be avoided. It is likely that women who choose to enter treatment are quite different from those who make no attempt to enter treatment.

## Conclusion

This study suggests that, for a sample of treatment-seeking women with histories of substance abuse, withholding access to basic needs, positive affiliations, and normal social roles does not reduce the propensity to relapse. Further, this study suggests support for Braithwaite's [18-20,22] theory that treating people in stigmatizing and punitive manners may actually increase their propensity to continue with substance-abusing and illegal behaviors.

The findings from this study suggest the need to replace punitive policies (e.g., withholding financial assistance) toward women who have histories of substance abuse with policies that allow these women to assume roles of responsibility such as work, education, and parenting. Strategies that support individuals seeking to develop or resume pro-social lives have potential positive implications for both individuals and society. At a minimum, policymakers should consider other factors besides past substance abuse and criminal behaviors when making decisions about how to treat women with histories of substance abuse.

Because the present study suggests that policies of stigmatization and punishment are not likely to reduce illegal behaviors, policymakers should consider radical changes in the approach to women with substance abuse problems, providing support and a path to a pro-social lifestyle that includes normal responsibilities. Continuing to stigmatize and punish substance-abusing women is likely to result in their further alienation, promoting rather than extinguishing drug abuse and criminal behaviors.

## Competing interests

The author(s) declare that they have no competing interests.

## Authors' contributions

NV is the sole author for this publication and has completed this work without substantive intellectual contributions of others.
